# The Mechanism of Ethical Leadership’s Influence on Nurses’ Voice Behavior: An Empirical Study Based on a Chain Mediation Model

**DOI:** 10.1155/jonm/7749514

**Published:** 2026-03-27

**Authors:** Jun-Tong Jing, Zheng-Yi Ma, Dong-Run Liu, Hang-Na Qiu, Yong-Kang Fu, Jie Liu, Chao-Ran Chen

**Affiliations:** ^1^ Nursing Department of Huaihe Hospital, Henan University, Kaifeng, China, henu.edu.cn; ^2^ Institute of Nursing and Health, School of Nursing and Health, Henan University, Kaifeng, China, henu.edu.cn

**Keywords:** ethical leadership, nurses, organizational commitment, psychological contract, voice behavior

## Abstract

**Background:**

Nurses’ voice behavior plays an irreplaceable role in optimizing nursing practices, improving healthcare quality, and fostering organizational innovation. However, existing research on the influencing factors and underlying mechanisms of nurses’ voice behavior remains limited and requires further exploration.

**Objective:**

This study aims to examine the relationship between ethical leadership and nurses’ voice behavior and further investigate the potential mediating roles of psychological contract and organizational commitment.

**Methods:**

From August to October 2024, a questionnaire survey was conducted, collecting valid data from 1357 nurses in China. The survey included the Ethical Leadership Scale, Psychological Contract Scale, Organizational Commitment Scale, and Voice Behavior Scale. Data were statistically analyzed using SPSS 25.0, and structural equation modeling (SEM) was performed using Amos 26.0 to examine the mediating roles of psychological contract and organizational commitment in the relationship between ethical leadership and nurses’ voice behavior.

**Results:**

Ethical leadership, psychological contract, organizational commitment, and voice behavior were significantly correlated (*p* < 0.001). Ethical leadership had a direct impact on voice behavior (effect = 0.360, 95% CI = [0.319, 0.399]). Psychological contract and organizational commitment played partial mediating roles in the relationship between ethical leadership and voice behavior (effect = 0.101, 95% CI = [0.086, 0.118], and effect = 0.010, 95% CI = [0.004, 0.016]). Additionally, a significant chain mediation effect of psychological contract and organizational commitment was found in the relationship between ethical leadership and voice behavior (effect = 0.024, 95% CI = [0.018, 0.031]), accounting for 4.848% of the total effect (0.024/0.495).

**Conclusion:**

Ethical leadership not only directly promotes nurses’ voice behavior but also indirectly enhances this effect through the chain mediation of psychological contract and organizational commitment. Strengthening ethical leadership can optimize nurses’ psychological contracts and enhance their organizational commitment, thereby effectively stimulating voice behavior.

**Implications for Nursing Management:**

Hospital administrators should recognize the critical role of ethical leadership in nursing management and adopt multilevel strategies to foster a positive organizational climate, optimize nurses’ psychological contracts, and strengthen their organizational commitment. These efforts will encourage nurses to actively voice their opinions, thereby enhancing team effectiveness and stability.

## 1. Introduction

In the context of the rapid development and transformation of the global healthcare system, medical organizations are facing multiple challenges such as population aging, shifts in the disease spectrum, and innovations in medical technology [1]. Healthcare institutions must not only ensure the quality of nursing care and service efficiency but also continuously advance organizational improvement and innovation [2]. In this process, the active participation and knowledge contributions of frontline employees have become increasingly important. As the largest professional group and the one in closest contact with patients, nurses’ experiences and insights are irreplaceable for organizational learning and service innovation [3]. Nurse voice behavior refers to an extra‐role behavior in which nurses proactively put forward opinions and suggestions with the aim of improving nursing practice and organizational operations [4]. Such behavior not only contributes to enhancing nursing quality and patient safety but also strengthens nurses’ professional beliefs and team cohesion [5]. However, constrained by the high‐pressure environment and hierarchical management culture prevalent in the healthcare industry, nurses’ voice behaviors are often difficult to fully exert [6]. Existing studies have shown that the factors influencing voice behavior mainly involve three levels: individual, work environment, and organizational management [7]. Therefore, how to effectively stimulate nurses’ enthusiasm and willingness to voice under multiple pressures and complex contexts has become an urgent issue in nursing management practice.

## 2. Background

In the field of organizational behavior research, ethical leadership is widely regarded as an important factor influencing employees’ proactivity and organizational behavior [8, 9]. However, how it functions in high‐pressure nursing contexts to affect voice behavior still lacks systematic explanation. Ethical leadership is a leadership style that conveys moral norms through personal conduct and interpersonal interactions [10], emphasizing fairness, transparency, and care, thereby enhancing employees’ psychological safety and organizational identification [11]. According to social learning theory [12], individuals adjust their behaviors by observing and learning from role models. In organizational settings, leaders’ demonstration effects provide behavioral references and value signals to subordinates [13]. When nursing managers display integrity, fairness, and respect for opinions, nurses perceive that “speaking up is both safe and valued,” making them more likely to overcome risk concerns and voice proactively [14]. Previous studies have also confirmed that ethical leadership can stimulate employees’ positive behaviors, including voice and organizational citizenship behavior [15]. Therefore, this study hypothesizes that ethical leadership has a significant positive effect on nurse voice behavior (Hypothesis 1).

However, relying on ethical leadership alone is not sufficient to fully explain nurses’ voice behavior under high‐pressure conditions. Psychological contract refers to employees’ subjective perceptions of organizational commitments, obligations, and expectations, reflecting exchange expectations based on reciprocity [16]. In nursing practice, voice is often a resource‐intensive behavior, requiring time, energy, and emotional investment, whereas a stable psychological contract can serve as a key reciprocal resource, providing nurses with necessary support and compensation [17]. Through an effective short‐term resource supplementation mechanism, the high costs of voice can be buffered, making nurses more willing to take potential risks. Ethical leadership, through integrity‐based demonstration and fair, transparent management practices, reinforces nurses’ trust and sense of security in the organization [18], thereby consolidating the psychological contract [19]. Compared with other psychological‐level mediators such as job satisfaction or psychological safety, the psychological contract better captures the dynamic balance between resource consumption and supplementation and was thus prioritized as a key mechanism [20, 21]. Previous studies have confirmed the close association between psychological contract and voice [22], but most of them focused on individual motivation while neglecting the tension between resource depletion and supplementation [3]. Therefore, this study hypothesizes that the psychological contract mediates the relationship between ethical leadership and nurse voice behavior (Hypothesis 2).

After revealing how the psychological contract helps nurses balance short‐term resource investment, this study further emphasizes the role of organizational commitment in explaining nurses’ sustained voice behavior over the long term. Organizational commitment refers to employees’ long‐term loyalty and sense of responsibility to the organization, reflecting intrinsic motivation based on emotional dependence and value identification [23]. Unlike the psychological contract, which focuses on short‐term resource supplementation grounded in reciprocal exchange, organizational commitment highlights a stable and enduring psychological bond that encourages nurses to remain engaged and to voice proactively even under high‐pressure conditions [24]. According to self‐determination theory [25], when basic needs for belonging, autonomy, and competence are fulfilled, employees’ commitment levels significantly increase. Ethical leadership, by demonstrating fairness, providing care and support, and offering development opportunities, can meet these needs, thereby enhancing nurses’ organizational commitment [13, 26], which in turn translates into sustained voice behavior [27]. Previous studies have confirmed a significant positive relationship between organizational commitment and voice behavior [28], but most did not systematically analyze how leadership style shapes this pathway [29]. Therefore, this study hypothesizes that organizational commitment mediates the relationship between ethical leadership and nurse voice behavior (Hypothesis 3).

It is worth noting that psychological contract and organizational commitment are interrelated in the formation of nurses’ proactive behaviors [30]. Loes and colleagues also pointed out that the fulfillment of psychological contracts can effectively strengthen employees’ organizational commitment, particularly with a pronounced effect on the dimension of affective commitment [31]. According to psychological contract theory [32], when organizations fulfill their promises and provide support, nurses’ psychological contracts are reinforced, which in turn elevates their organizational commitment. This process is also consistent with conservation of resources theory [33]: the psychological contract can be regarded as a critical psychological resource acquired by nurses, while organizational commitment represents the motivational sedimentation of such accumulated resources. This forms a mechanism of “resource input ⟶ motivational accumulation ⟶ behavioral output,” which provides intrinsic motivation for nurses to sustain voice behavior under high‐pressure conditions. However, existing studies often examined psychological contract and organizational commitment separately, overlooking their sequential effects [34]. Therefore, we hypothesize that ethical leadership influences nurse voice behavior through the sequential mediation of psychological contract and organizational commitment (Hypothesis 4).

**FIGURE 1:**
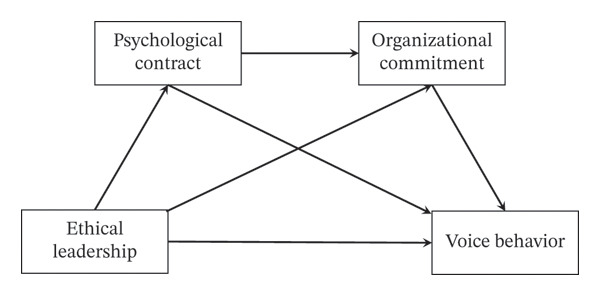
Theoretical framework.

In summary, by integrating multiple theoretical perspectives—social learning theory, self‐determination theory, psychological contract theory, and conservation of resources theory—this study proposes a “demonstration–resource gain–behavioral return” chain model (Figure 1) to explore the mechanisms through which ethical leadership influences nurse voice behavior, with a particular focus on testing the mediating roles of psychological contract and organizational commitment. This provides a pathway reference for nursing management practice.

## 3. Methods

### 3.1. Study Design

This study employed a cross‐sectional correlational design to explore the relationships among ethical leadership, psychological contract, organizational commitment, and voice behavior. Additionally, a structural equation model (SEM) was constructed to examine the mediating roles of psychological contract and organizational commitment. This study followed the STROBE guidelines.

### 3.2. Setting, Participants, and Sample

A multistage sampling method was used to ensure the representativeness and scientific rigor of the study sample. First, Henan Province was divided into five regions: East Henan, West Henan, South Henan, North Henan, and Central Henan, each representing different levels of medical resource allocation and nursing practice characteristics. In the first stage, three regions (Central Henan, East Henan, and South Henan) were randomly selected. In the second stage, two cities were randomly selected from each chosen region (e.g., Zhengzhou and Luohe, Kaifeng and Shangqiu, and Xinyang and Nanyang). In the third stage, one tertiary general hospital was randomly selected from each selected city, resulting in a total of six hospitals as research sites. The inclusion criteria for participants were as follows: (1) registered nurses currently employed in the hospital, (2) at least one year of work experience, and (3) voluntary participation with informed consent. The exclusion criteria included (1) intern nurses or nursing students and (2) incomplete or invalid questionnaire responses.

The sample size was calculated using the formula N = 4*U* *α* ^2^ *S* ^2^/*δ* ^2^. Based on a preliminary study, the standard deviation (S) of the dependent variable (voice behavior) was 0.89, and the allowable error was set at 0.1, with *α* = 0.05. The initial sample size was estimated as *N* = 4 × 1.962 × 0.89^2^/0.1^2^ ≈ 1217. To account for sampling errors and invalid questionnaires, the research team increased the sample size to 1400. A total of 1371 questionnaires were collected, with a response rate of 97.92%. After excluding 14 invalid questionnaires (e.g., incomplete responses or identical answers for all items), 1357 valid questionnaires remained, yielding an effective response rate of 96.93%.

### 3.3. Data Collection and Ethical Considerations

Data collection was conducted between August and October 2024. Before the formal survey, the research team provided standardized training to investigators to ensure consistency in data collection. After obtaining written approval from the hospital administration and nursing departments, study notifications were distributed to eligible nurses through the hospital’s internal communication system. The questionnaire was completed anonymously using a self‐administered format. Each participant received a sealed envelope containing the questionnaire, an informed consent form, and detailed study instructions. Before completing the questionnaire, researchers provided a thorough explanation of the study’s purpose, significance, data confidentiality, and participants’ rights. Participants were informed that their participation was entirely voluntary and that they could withdraw at any time without any consequences. All participants provided written informed consent prior to completing the questionnaire. During data collection, researchers did not provide any hints or inducements to respondents.

All collected data were strictly confidential and used solely for research purposes. This study was reviewed and approved by the Biomedical Research Ethics Subcommittee of Henan University (approval no.: HUSOM2023‐478) and adhered to the ethical principles outlined in the Declaration of Helsinki. Through multistage sampling, rigorous sample size calculation, standardized data collection procedures, and comprehensive ethical review, this study ensured methodological rigor and data reliability, thereby providing a solid foundation for the scientific validity and generalizability of the findings.

### 3.4. Measurement Instruments

#### 3.4.1. Ethical Leadership

The Ethical Leadership Scale (ELS) was developed by Brown et al. [10] and later translated into Chinese by Huang et al. [35, 36]. The scale demonstrated a Cronbach’s α coefficient of 0.95 and has been widely applied in the nursing field [36]. The ELS is a unidimensional instrument consisting of 10 items, using a 5‐point Likert scale (1 = *strongly disagree* and 5 = *strongly agree*), with a total score ranging from 10 to 50. Higher scores indicate higher levels of ethical leadership. In this study, the Cronbach’s α coefficient of the scale was 0.97.

#### 3.4.2. Psychological Contract

The Psychological Contract Scale was translated into Chinese by Liu et al. [37] as a psychometric instrument. The scale consists of two dimensions—performance excellence and loyalty—with a total of 8 items. It uses a 5‐point Likert scale (1 = *strongly disagree* and 5 = *strongly agree*), with higher scores indicating higher levels of psychological contract. The Cronbach’s α coefficient of the scale was 0.81, and it has been validated among Chinese nurses, demonstrating good reliability and validity. In the present study, the Cronbach’s α coefficient of the scale was 0.97.

#### 3.4.3. Organizational Commitment

The Organizational Commitment Questionnaire (OCQ), originally developed by Meyer et al. [38], was adopted in this study. The scale demonstrated a Cronbach’s α coefficient of 0.85 and has been widely applied among Chinese nurses [39, 40]. It consists of three dimensions—affective commitment, normative commitment, and continuance commitment—with a total of 18 items. Responses are rated on a 5‐point Likert scale, with higher scores indicating stronger organizational commitment. In the present study, the Cronbach’s α coefficient of the scale was 0.96.

#### 3.4.4. Voice Behavior

The 10‐item Chinese version of the voice behavior scale was developed by Liang et al. [41]. The scale consists of two dimensions with a total of 10 items, measuring Promotive Voice Behavior (5 items) and Prohibitive Voice Behavior (5 items). It adopts a 5‐point Likert scale (1 = *strongly disagree* and 5 = *strongly agree*), with higher scores indicating higher levels of nurse voice behavior. The Cronbach’s α coefficient of the scale was 0.93. In the present study, the Cronbach’s α coefficient of the scale was 0.95.

### 3.5. Data Analysis

Data analysis was conducted using IBM SPSS 25.0 and AMOS 26.0. First, descriptive statistics such as frequencies and percentages were used to summarize participants’ demographic characteristics. Normality tests were performed for continuous variables. If ethical leadership, psychological contract, organizational commitment, and voice behavior followed a normal distribution, Pearson correlation analysis was used to examine the relationships between these variables; otherwise, Spearman correlation analysis was applied. Second, AMOS 26.0 was used to construct a SEM, in which ethical leadership was the independent variable, voice behavior was the dependent variable, and psychological contract and organizational commitment were mediating variables. Following the recommendations of Hair et al., SEM analysis was divided into two parts: in the measurement model analysis, the convergent validity, discriminant validity, and construct reliability of latent variables were tested; in the structural model analysis, path analysis and hypothesis testing were conducted. Finally, a bootstrapping method with 5000 resamples was employed to assess the multiple mediation effects and bias‐corrected confidence intervals (CIs). A 95% CI that did not include zero indicated a significant mediation effect. A two‐tailed *p* value of < 0.05 was considered statistically significant.

## 4. Results

### 4.1. Common Method Bias Test

First, Harman’s single‐factor test was conducted to examine common method bias. The results showed that four factors had eigenvalues greater than 1, with the first factor accounting for 48.75% of the total variance, which is below the critical threshold of 50% [42], indicating that no significant common method bias existed. Furthermore, following the recommendations of Kock [43] and Lynn [44], we tested for potential common method bias using the variance inflation factor (VIF). The results revealed that all measurement items had VIF values below 2, suggesting that common method bias had no substantial impact on the study’s findings. Therefore, the data in this study demonstrated good reliability and validity, and no serious common method bias was detected.

### 4.2. Demographic Characteristics of Participants

A total of 1357 young nurses participated in this study. The majority of participants were under the age of 30. Among them, 1005 (74.1%) were female and 352 (25.9%) were male. The largest age group was 24–29 years (45.2%). Most participants were unmarried (79.8%), and the majority held a bachelor’s degree (67.4%). The most common monthly income level was ≤ 6000 RMB (42.2%), with the majority working 7‐8 h per day (59.8%) and having 5–8 night shifts per month (44.7%). Detailed demographic information is presented in Table 1.

**TABLE 1:** Characteristics of participants (*N* = 1357).

Demographics	*N* (%)
Gender	
Man	352 (25.9)
Female	1005 (74.1)
Age	
< 24 years old	583 (43.0)
24–29 years old	613 (45.2)
> 29 years old	161 (11.9)
Marital status	
Unmarried	1083 (79.8)
Married	264 (19.5)
Divorced	10 (0.7)
highest education	
College or below	273 (20.1)
Bachelor degree	914 (67.4)
Master degree or above	170 (12.5)
Average monthly income	
≤ 6000	572 (42.2)
6000–8000	517 (38.1)
8000–10,000	166 (12.2)
≥ 10,001	102 (7.5)
The average daily working time was	
< 7 h	352 (25.9)
7‐8 h	811 (59.8)
≥ 9 h	194 (14.3)
The average number of night shifts per month	
≤ 4 times	485 (35.7)
5–8 times	607 (44.7)
≥ 9 times	265 (19.5)

### 4.3. Measurement Model Assessment

The measurement model was evaluated using standardized factor loadings, Cronbach’s *α*, composite reliability (CR), and average variance extracted (AVE). In general, when factor loadings exceed 0.60, Cronbach’s α and CR are above 0.70, and AVE is greater than 0.50, the scale is considered to demonstrate good reliability and convergent validity [45]. As shown in Table 2, the standardized loadings of all measurement indicators ranged from 0.92 to 0.95, well above the recommended threshold. The Cronbach’s α and CR values of all latent variables were greater than 0.93, indicating strong internal consistency. Meanwhile, the AVE values ranged from 0.78 to 0.90, exceeding the 0.50 standard, which further supports convergent validity. Therefore, the measurement model in this study demonstrated good reliability and convergent validity, providing a robust foundation for subsequent structural model analysis.

**TABLE 2:** Standardized loadings and reliabilities.

Contract	Indicators	Standardized loadings	Cronbach’s α	CR	AVE
Psychological Contract	PE	0.95	0.97	0.95	0.90
	Loyalty	0.95			

Organizational Commitment	AC	0.92	0.97	0.95	0.86
	NC	0.95			
	CC	0.92			

Voice behavior	PVB	0.94	0.96	0.93	0.87
	IVB	0.93			

Ethical Leadership			0.95	0.97	0.78

### 4.4. Correlation Analysis and Significance Testing

Since ethical leadership, psychological contract, organizational commitment, and voice behavior followed a normal distribution; Pearson correlation analysis was used to examine their relationships, as shown in Table 3. Voice behavior had a moderately high mean score (36.753 ± 7.150) and showed significant positive correlations with ethical leadership (*r* = 0.657, *p* < 0.01), psychological contract (*r* = 0.695, *p* < 0.01), and organizational commitment (*r* = 0.563, *p* < 0.01). Ethical leadership was positively correlated with psychological contract (*r* = 0.559, *p* < 0.01) and organizational commitment (*r* = 0.416, *p* < 0.01). Additionally, psychological contract and organizational commitment were significantly positively correlated (*r* = 0.567, *p* < 0.01).

**TABLE 3:** Descriptive statistics and correlations among the study variables (*N* = 1357).

	**EL**	**PC**	**OC**	**VB**	**Mean**	**Standard deviation**

EL	1.000				34.717	8.280
PC	0.559^∗∗^	1.000			29.051	6.957
OC	0.416^∗∗^	0.567^∗∗^	1.000		65.416	13.537
VB	0.657^∗∗^	0.695^∗∗^	0.563^∗∗^	1.000	36.753	7.150

Abbreviations: EL, ethical leadership; OC, organizational commitment; PC, psychological contract; VB, voice behavior.

^∗∗^ *p* < 0.01 (two‐tailed).

### 4.5. Mediation Effect Analysis

This study constructed a SEM with ethical leadership as the independent variable, psychological contract and organizational commitment as mediating variables, and voice behavior as the dependent variable. The results indicated that the model exhibited a good fit, as detailed in Table 4.

**TABLE 4:** Structural equation model fitting index.

Model	CMIN/DF	RMSEA	GFI	CFI	TLI	IFI
Recommended value	< 5	< 0.08	> 0.90	> 0.90	> 0.90	> 0.90
Mediation model	3.082	0.039	0.992	0.997	0.995	0.997

*Note:* CMIN/DF, the chi‐square divided by degrees of freedom.

Abbreviations: CFI, the comparative fit index, GFI, the goodness‐of‐fit index; IFI, incremental fit index; RMSEA, root means square error of approximation; TLI, Tucker–Lewis Index.

**FIGURE 2:**
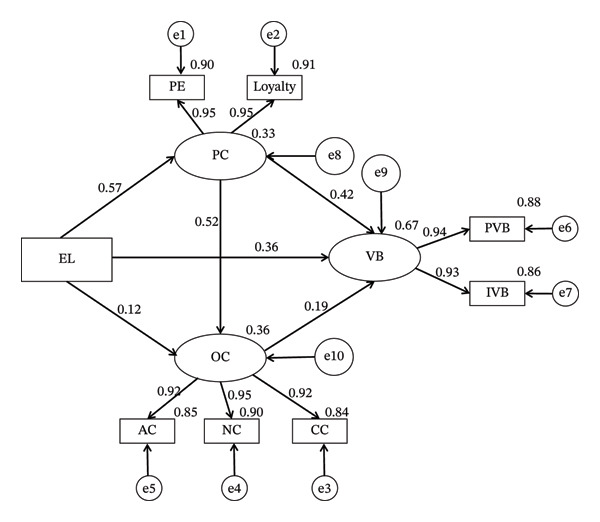
Path analysis diagram of ethical leadership, psychological contract, organizational commitment, and voice behavior. EL: ethical leadership; PC: psychological contract; OC: organizational commitment; VB: voice behavior; PE: performance excellence; AC: affective commitment; NC: normative commitment; CC: continuance commitment; PVB: promotive voice behavior; IVB: prohibitive voice behavior.

Based on Figure 2, ethical leadership has a significant direct positive effect on psychological contract (*β* = 0.573, *p* < 0.001), voice behavior (*β* = 0.360, *p* < 0.001), and organizational commitment (*β* = 0.122, *p* < 0.001). Psychological contract has a significant direct positive effect on organizational commitment (*β* = 0.523, *p* < 0.001) and voice behavior (*β* = 0.419, *p* < 0.001). Additionally, organizational commitment also has a significant direct positive effect on voice behavior (*β* = 0.191, *p* < 0.001).

Finally, we applied the percentile bootstrap method with 5000 resamples and a 95% CI to further investigate the mediating roles of psychological contract and organizational commitment in the relationship between ethical leadership and voice behavior. The results showed that both psychological contract and organizational commitment exerted partial mediating effects. Specifically, the mediating effect of psychological contract was 0.101, accounting for 20.4% of the total effect; the mediating effect of organizational commitment was 0.010, with a relatively small proportion. In addition, psychological contract and organizational commitment jointly produced a sequential mediation effect, accounting for 4.848% of the total effect. Details are presented in Table 5.

**TABLE 5:** Bootstrap analysis of the mediating model.

Effects	Paths	Effect	Bootstrap SE	Bootstrapping 95% CI
Total effect	EL ⟶ VB	0.495	0.018	0.458 to 0.529
Direct effect	EL ⟶ VB	0.360	0.020	0.319 to 0.399

Indirect effect	EL ⟶ PC ⟶ VB	0.101	0.008	0.086 to 0.118
	EL ⟶ OC ⟶ VB	0.010	0.003	0.004 to 0.016
	EL ⟶ PC ⟶ OC ⟶ VB	0.024	0.003	0.018 to 0.031

Total indirect effect	Total indirect effect	0.135	0.008	0.119 to 0.152

## 5. Discussion

This study found that ethical leadership had a significant direct positive effect on nurse voice behavior, which confirmed Hypothesis 1. This result is consistent with the findings of Zhou and Zhang, who also emphasized that ethical leadership can effectively promote nurses’ proactive work behaviors within organizations [46]. However, unlike their study, the present research not only confirmed this direct effect but also situated it within the broader framework of “demonstration–resource gain–behavioral return,” highlighting the foundational role of ethical leadership as the “demonstration” mechanism in this model. The potential mechanism underlying this result can be explained through psychological safety. Ethical leaders, by demonstrating fairness, justice, and integrity in the workplace, enable nurses to feel respected and supported, thereby reducing concerns about the potential risks of voicing opinions [47]. Previous research has also shown that when employees experience higher levels of psychological safety within organizations, they are more willing to express suggestions and ideas, which in turn enhances voice behavior [48]. In the Chinese cultural context, the positive impact of ethical leadership is particularly salient [49]. The qualities emphasized by ethical leadership—integrity, fairness, and benevolence—resonate strongly with the Confucian concept of *ren* (benevolence) [50]. Through long‐term interactions, nurses are more likely to perceive such leadership behaviors as recognition and respect for their own value by the organization, thereby strengthening their sense of identification and responsibility toward the organization [51]. This cultural congruence further reinforces nurses’ willingness to voice under high‐pressure work environments. Based on these findings, nursing managers should pay close attention to the cultivation and practice of ethical leadership in management practice.

The results of this study also show that ethical leadership not only directly influences nurses’ voice behavior but also has an indirect effect through the partial mediating role of psychological contract, thus confirming Hypothesis 2. Nurses who perceive higher levels of ethical leadership are more likely to establish positive psychological contracts, a finding consistent with the research of Pakizekho and colleagues [26]. This phenomenon can be explained from an attribution process perspective. When nurses perceive their managers’ integrity, fairness, and care, they are more likely to attribute these behaviors to the organization’s recognition and support rather than seeing them as mere management strategies [52]. This attribution process enhances nurses’ identification with the organization, making them more willing to fulfill their psychological contract and give back to the organization. Furthermore, the study found that psychological contract had a significant positive impact on nurse voice behavior, consistent with prior research [53]. According to empowerment theory, when nurses perceive fair treatment and professional support from the organization, their sense of autonomy and influence increases, thereby enhancing their willingness to engage in voice behavior [54]. Compared with passively following organizational rules, nurses with stronger psychological contracts are more inclined to actively contribute their own insights to promote organizational improvement and innovation [55].

Notably, our findings indicated that among all mediating variables, the psychological contract exhibited the largest effect size (accounting for 20.4% of the total effect), underscoring its central position in the “demonstration–resource gain–behavioral return” framework and highlighting its importance as a “resource supplementation” mechanism. This finding extends prior studies that predominantly focused on individual motivation [56], offering a new theoretical lens for understanding how leadership behaviors shape nurse voice.

This study found that organizational commitment partially mediated the relationship between ethical leadership and nurse voice behavior, thereby confirming Hypothesis 3; this result is consistent with prior findings [57]. At the mechanistic level, ethical leadership, through fair and transparent interactions, provides nurses with consistent value orientations and supportive cues, thereby enhancing their perceived organizational support and value congruence, which in turn strengthens organizational commitment—particularly affective commitment [58, 59]. This process reduces the relational risks associated with voicing, fostering a climate of psychological safety [60]. At the same time, according to social identity theory, nurses are more likely to internalize organizational goals as part of their self‐identity within such a climate, thus maintaining voice behavior with a stronger sense of responsibility and belonging [60]. Moreover, from a work motivation perspective, ethical leadership meets nurses’ needs for relatedness and competence, allowing organizational commitment to emerge as a more autonomous and enduring motivational force [61]. This shift transforms commitment from “compliance‐based involvement” to “improvement‐oriented involvement,” thereby promoting voice behaviors aimed at quality improvement [62]. From the perspective of effect sizes, the mediating effect of organizational commitment was smaller than that of the psychological contract. Given its theoretical connotation as a more stable emotional bond, we interpret organizational commitment as operating primarily on the temporal dimension, supporting the maintenance and recurrence of voice behaviors over time [24]. This explanation aligns with commitment theory, which emphasizes the role of affective commitment in sustaining voluntary behaviors [63], and is consistent with the relatively small but significant indirect effect observed in the “ethical leadership ⟶ organizational commitment ⟶ voice” pathway. Accordingly, we infer that in the “demonstration–resource gain–behavioral return” framework, organizational commitment plays a bridging role by transforming relational cues triggered by leadership into more enduring motivations based on identity and responsibility. It should be noted, however, that given the cross‐sectional design and reliance on self‐reported measures, these temporal and mechanistic explanations require further testing through longitudinal or multi‐wave, multi‐source data.

The main innovation of this study lies in uncovering the sequential mediating effect of psychological contract and organizational commitment in the relationship between ethical leadership and nurse voice behavior. Specifically, while prior studies have primarily examined the associations of psychological contract or organizational commitment with proactive behaviors separately [34], this study demonstrates that the two are not independent and parallel mechanisms but are sequentially connected through a “psychological contract ⟶ organizational commitment” relay process, jointly transmitting the influence of leadership on voice. At the mechanistic level, the strengthening of psychological contract reflects nurses’ consistent experiences of “fulfilled promises, procedural and distributive fairness, and developmental prospects,” which can be regarded as critical psychological resources obtainable and accumulable in high‐pressure nursing contexts [32, 33]. When nurses interpret organizational behaviors as positive signals of respect and support [64], their trust and value congruence increase, and a reinforced psychological contract further externalizes into stronger organizational commitment [30]. Organizational commitment, in turn, is not merely a simple return of resources but rather a process of motivational consolidation and persistence: it transforms accumulated trust and support into a more stable sense of identity and responsibility, thereby enhancing the sustainability and recurrence of voice behaviors [34]. Moreover, related research suggests that proactive behaviors are shaped not only by individual traits or short‐term motivation but also by systematic organizational and contextual factors [65]. Accordingly, relying solely on individual‐level efforts may be insufficient to enhance voice; more crucial are organizational‐level institutional support and contextual interventions. Such measures include strengthening the consistency and demonstrative role of ethical leadership, improving mechanisms for promise fulfillment and feedback, ensuring access to resources and developmental opportunities, and advancing participatory governance. By consolidating psychological contract and embedding it into stable organizational commitment, these organizational interventions can more effectively foster and sustain nurses’ voice behavior. It should be noted that the mediation effects observed in this study were relatively small. This may be attributable to the limited work tenure of the participants, as the sample consisted predominantly of early‐career nurses whose psychological contracts and organizational commitments are still developing. Such developmental immaturity may attenuate the strength of the indirect effects, leading to modest mediation estimates. Therefore, the mechanisms identified in this study may primarily reflect the behavioral patterns of younger, less‐tenured nurses. Future research should examine these mediation pathways in samples with more diverse and extended work tenure structures to enhance the explanatory power and generalizability of the findings.

## 6. Theoretical Contributions

This study extends the existing theoretical framework in several ways and provides new evidence from the Chinese nursing context. First, it proposed and validated a sequential mediation model that integrates psychological contract and organizational commitment into the “demonstration–resource gain–behavioral return” pathway. This compensates for prior studies that examined these two constructs separately and offers a new perspective for understanding the mechanisms through which ethical leadership operates under high‐pressure nursing conditions. Second, the findings not only corroborate the demonstration effect of leadership emphasized in social learning theory but also align with psychological contract theory and self‐determination theory in explaining proactive employee behaviors. Moreover, the results further enrich the application of conservation of resources theory in nursing contexts by highlighting the dynamic linkages among resource input, motivational consolidation, and behavioral output. Finally, by focusing on a sample of Chinese nurses, this study reveals how individual–organization interactions shape nurse voice behavior. This not only expands the applicability of organizational behavior theories in the nursing field but also provides empirical support for cross‐cultural comparative studies on leadership and voice behavior in the international academic community.

## 7. Limitations

This study has several limitations. First, it is a cross‐sectional design, which only explores the relationships between variables at a single time point. Future research could use longitudinal studies or other methods to explore the dynamic development and related mechanisms of these variables. Second, data collection relied primarily on self‐administered questionnaires. Although measures were taken during the design and implementation stages to minimize common method bias, such bias cannot be completely eliminated. Future research could incorporate multisource data or combine ratings from different evaluators to further enhance the objectivity and robustness of the findings. Third, the generalizability of our findings may be limited as the sample predominantly consisted of early‐career nurses, whose behavioral patterns may not represent the entire nursing workforce. Future research should consider work tenure as a potential moderating factor to address this limitation. Finally, the participants in this study were all nurses from Henan Province. Given the potential regional differences, the generalizability of the findings may be limited. Future research could consider expanding the sample size and geographical scope to strengthen the generalizability of the results.

## 8. Conclusion

This study confirmed the significant positive effect of ethical leadership on nurse voice behavior and revealed the sequential mediating mechanisms of psychological contract and organizational commitment. The findings expand the theoretical framework of nurse voice research and enrich the applicability of ethical leadership in high‐pressure nursing contexts. At the same time, the study provides practical guidance for nursing management, suggesting that managers should strengthen ethical leadership, consolidate psychological contracts, and enhance organizational commitment in order to better stimulate nurses’ willingness to voice.

## 9. Implications for Nursing Management

The findings of this study provide targeted recommendations for nursing management practice in China and offer implications for similar contexts. First, enhancing ethical leadership: the results demonstrate that ethical leadership has both direct and indirect positive effects on nurse voice behavior. Nursing managers should focus on improving their own ethical qualities and managerial capabilities, for example, by organizing ethics‐oriented leadership training and incorporating fairness and transparency into managerial performance assessments, thereby creating a safe, trustworthy, and supportive work environment for nurses. Second, strengthening the psychological contract: given that the psychological contract accounted for the largest mediating effect between ethical leadership and voice behavior (20.4% of the total effect), nursing managers should prioritize enhancing nurses’ trust in the organization by fulfilling promises and improving promotion and feedback mechanisms. These practices can not only reduce nurses’ concerns when voicing but also effectively stimulate their willingness to engage in organizational improvement. Third, enhancing organizational commitment: although its effect size was relatively smaller, organizational commitment plays a vital role in sustaining nurses’ long‐term voice behavior. Managers can implement a dual‐track incentive system—combining career development support with emotional care—to strengthen nurses’ loyalty and sense of responsibility toward the organization, thereby promoting sustained voice. Finally, providing management support in high‐pressure contexts: the results revealed that even under high‐pressure nursing conditions, ethical leadership can still stimulate nurse voice. Managers should, therefore, provide resource support and emotional care during peak periods of work stress to further buffer the adverse impact of pressure on voice behavior.

## Funding

This work was supported by the Soft Science Project of Henan Provincial Department of Science and Technology (Grant no. 242400410523); the “Mingde” Online Ideological and Political Studio of Henan University; and the Henan Medical Science and Technology Research Project (Grant no. RKX202402028).

## Disclosure

The funders had no role in the study design, data collection, analysis, interpretation, or manuscript preparation.

## Conflicts of Interest

The authors declare no conflicts of interest.

## Data Availability

Data used to support the findings of this study are available on request from the corresponding author.
